# Chemical and Rheological Evaluation of Aged Lignin-Modified Bitumen

**DOI:** 10.3390/ma12244176

**Published:** 2019-12-12

**Authors:** Yi Zhang, Xueyan Liu, Panos Apostolidis, Wolfgang Gard, Martin van de Ven, Sandra Erkens, Ruxin Jing

**Affiliations:** 1 School of Highway, Chang’an University, Xi’an 710064, China; 2 Faculty of Civil Engineering and Geosciences, Delft University of Technology, Stevinweg 1, 2628 CN Delft, The Netherlands; P.Apostolidis@tudelft.nl (P.A.); W.F.Gard@tudelft.nl (W.G.); M.F.C.vandeVen@tudelft.nl (M.v.d.V.); S.M.J.G.Erkens@tudelft.nl (S.E.); R.Jing@tudelft.nl (R.J.)

**Keywords:** lignin, bitumen, aging, microstructure, chemistry, rheology

## Abstract

As bitumen oxidizes, material stiffening and embrittlement occur, and bitumen eventually cracks. The use of anti-oxidants, such as lignin, could be used to delay oxidative aging and to extend the lifetime of asphalt pavements. In this study, the chemical and rheological effect of lignin on bitumen was evaluated by using a single dosage organsolv lignin (10 wt.% dosage). A pressure aging vessel (PAV) was used to simulate the long-term aging process after performing the standard short-term aging procedure, and the lignin-modified bituminous binders were characterized by an environmental scanning electron microscope (ESEM), Fourier-transform infrared (FTIR) spectroscopy, and a dynamic shear rheometer (DSR). From the ESEM results, the uniform microstructure was observed, indicating that the addition of lignin did not affect the worm structure of bitumen. Based on the FTIR test results, lignin-modified bitumen showed that a lower number of carbonyl and sulfoxide compounds were generated after aging than for neat bitumen. Based on the linear amplitude sweep (LAS) results, the addition of lignin slightly reduced the fatigue life of bitumen. From the frequency sweep results, it showed that lignin in bitumen acts as a modifier since the physical interaction between lignin and bitumen predominantly affects the material rheology. Overall, lignin could be a promising anti-oxidant due to its economic and environmental benefits.

## 1. Introduction

Bitumen is a hydrocarbon residue produced from oil refining and comprises a plethora of different organic molecules causing its vulnerability to environmental conditions [1]. Currently, the rising cost of bitumen, together with the fact that asphalt production is one of the largest energy consumers, globally encourages the use of alternative systems to replace petroleum-based binders to enhance the quality of pavement materials. Therefore, the environmental concerns and the demand for developing long-lasting pavements drive the asphalt industry to assess the possible use of bio-based artificial made binders [2,3] or waste and easily available polymers in bitumen.

Lignin is the most abundant bio-based polymer that can be found in co-products of the wood industry making up about 20% to 25% of the dry mass of every plant [4]. The total amount of lignin present in the biosphere exceeds 300 billion tons and increases by approximately 20 billion tons every year [5]. Specifically, lignin is a type of complex organic polymers that contributes to forming the cell walls in plants. In addition, bitumen is composed of millions of different organic molecules, the utilization of lignin may be used to substitute partially petroleum-based binders that assist toward more sustainable development in the bitumen industry. Therefore, the utilization of lignin in bitumen, specially designed for pavements, attracted considerable attention in recent years [6,7,8,9,10,11,12,13,14,15,16,17].

One of the reasons that asphalt pavement cracks is the stiffening and embrittlement of the bitumen due to aging [1]. Based on previous studies, lignin shows oxidative aging resistance because of its radical scavenging activity and polyphenolic structure [18,19,20]. Recent research focused on the addition of lignin as a type of anti-oxidant to bitumen [7,8,9,10,11,12,13,14,15,16]; however, it is important to verify whether the lignin reacts with bitumen to improve the aging resistance of bitumen. Moreover, the microstructure of lignin–bitumen binders is not yet clear. Thus, an in-depth understanding of the effect of lignin on bitumen will help to optimize the technology of bio-based binders, leading to more environmentally friendly pavement materials. In this research, special emphasis was given on assessing the impact of wood lignin powder extracted by the organosolv method on the chemistry and rheology of bitumen after aging.

## 2. Objective and Approach

The objective of this study was to evaluate the compositional and rheological changes of lignin-modified bitumen due to aging. This study consists of three parts. In the first part, the microstructure of different materials was measured by an environmental scanning electron microscope (ESEM). In the second part, the chemical components of lignin and bitumen were characterized by Fourier-transform infrared (FTIR) spectroscopy, and, finally, the mechanical properties of various lignin-bitumen combinations were studied using a dynamic shear rheometer (DSR). Frequency sweep, linear amplitude sweep, and relaxation tests performed in DSR. A pressure aging vessel (PAV) was used to simulate the aging of bitumen.

Overall, this article was designed to achieve the following aims:

1. Examine the effect of lignin on bitumen performance. Also, few studies focused on the aging of lignin and, thus, the aged lignin was evaluated using ESEM and FTIR spectroscopy.
2. Assess the effect of aging on lignin-modified bitumen. ESEM, FTIR, and DSR tests were conducted on samples in order to explore the microstructural, chemical, compositional, and rheological changes of new binders.

## 3. Materials and Methods

### 3.1. Materials and Samples Preparation

A 70/100 pengrade bitumen was used in this study. The softening point of this bitumen was 47.5 °C. The wood lignin was a brown powder obtained from Chemical Point UG (Oberhaching, Germany). After extraction by the organosolv method, a content of 88% lignin was obtained. The density of the lignin was 1.3774 g/cm^3^, which was measured by a helium pycnometer test, and the specific surface area was 147.0593 m^2^/g, which was measured by a surface analyzing system (DVS Resolution). The physical properties of the lignin were measured after aging as well. The overall color of lignin particles became darker, the density was increased to 1.5029 g/cm^3^, and the specific surface area was decreased to 65.0475 m^2^/g. As mentioned in Reference [8], 10 wt.% lignin was added by substituting an equivalent amount of bitumen. An overview of the studied materials is provided in Table 1.

The mixing time and temperature of lignin in bitumen were determined and described elsewhere [10]. Lignin (10 wt.% of bitumen) was gradually added to the bitumen, and then the two materials were mixed by a high shear mixing device at 163 °C and a mixing rate of 3000 rpm. The mixing was continued for about 30 min until the bubbles disappeared.

According to the standard testing procedure (ASTM D 6521-19 [21]), PAV was used to simulate the long-term aging process of bitumen and performed after the standard short-term aging procedure. In this study, 50 ± 0.5 g bitumen was poured into a PAV pan to form a film with 3.2-mm thickness. Then, the PAV test was performed at a temperature of 100 °C under pressurized air at 2.10 MPa for 20 h.

### 3.2. Microstructural Morphology

The microstructural observations were conducted at room temperature with a Philips XL30 environmental scanning electron microscope (ESEM, Eindhoven, Netherlands) under an acceleration voltage of 20 keV, similar to Reference [22], with a spot size of 3.5 in a chamber at 1.0 Torr pressure in low vacuum and secondary electron detector mode. The magnification was varied from ×125 to ×250, ×500, and ×1000. The scanning method was 1.68 ms × 484 lines, and the integration was 16 frames. The time of exposure was gradually increased from 0.5 to 1, 3, 5, and 10 min. As the exposure time increases, the energy absorbed by the surface of the material increases, and the light components in bitumen, such as saturates and aromatics, mostly evaporate; thus, the internal structure is much easier to be observed.

**Figure 1:**
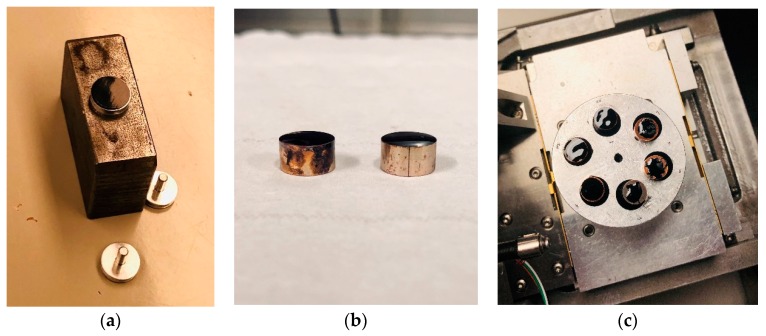
(**a**,**b**) Environmental scanning electron microscope (ESEM) samples on the holder, and (**c**) samples ready to be analyzed in ESEM.

Through the sample preparation for ESEM analyses, lignin and lignin-modified bitumen were placed on special sample holders as in Reference [22]. In particular, lignin was placed in an oven at 150 °C for 24 h to ensure drying before scanning. After attaching a small black sheet with adhesive on both sides of the plate, approximately 20 mg of lignin was poured onto an 8-mm-diameter sample holder (Figure 1a). The plate was tapped and vibrated to prevent powder build-up and to distribute it as evenly as possible on the black sheet for scanning. For the lignin-modified bitumen, the diameter and height of the sample holder cylinder were 9.20 mm and 5.20 mm, respectively (Figure 1b). The bitumen was placed in the oven to become liquid and flow easily on the holder After evenly stirring, a small amount of bitumen was dropped on the cylinder holder, and then the sample was placed back in the oven to set flat and uniformly (Figure 1c). It is important to protect the samples from dust or other impurities before ESEM scanning.

### 3.3. Chemical Characterization

FTIR is the most commonly used tool to detect the chemical compounds in bitumen [23] and lignin [18,24]. Different functional groups have a different light-absorption spectrum. Wavenumbers of typical bands of lignin and bitumen are listed in Table 2. In this study, attenuated total reflectance (ATR) FTIR was performed to collect spectral data of lignin and bitumen samples. The Spectrum 100 FTIR Perkin Elmer spectrometer with a single-point ATR fixture (Waltham, MA, USA) was used. The wavenumber ranged from 600 to 4000 cm^−1^ with a resolution of 4 cm^−1^. Before scanning, the lignin samples were dried at 140 °C for 30 min to remove any volatiles from samples. For the bitumen samples, the prism was cleaned with methylene chloride after each scan. Nine replicates per material were analyzed.

The functional group absorbance index (*AI*) was used for the main absorption bands of lignin to compare the changes of functional groups with the changes in spectra, and it was determined as follows:

(1) $$AI=\frac{A_{ab}}{\sum A},$$

where *A_ab_* is the integral area of absorption band *ab*, and ∑*A* is the sum of the integral areas of several characteristic functional group peaks. The range of chemical functional groups to be calculated and considered is summarized in Table 2.

Conventional aging indices of bitumen are the carbonyl (C=O) and sulfoxide (S=O) indices [25]. The effect of lignin as an anti-oxidant can be estimated by measuring the changes in the carbonyl and sulfoxide groups. Lignin is a combination of organic substances, and it contains carbonyl groups as well. The question of whether the aging of lignin has an impact on the aging index during the aging process should be strictly verified. Two aging indices were used to evaluate the anti-oxidation effect of lignin in bitumen, based on changes in carbonyl and sulfoxide groups, as follows:

(2) $$I_{C=o}=\frac{A_{S=O}}{\sum A},\;I_{S=o}=\frac{A_{S=O}}{\sum A},$$

where *A_C=O_* and *A_S=O_* are the integrated areas of carbonyl (C=O) and sulfoxide (S=O) groups, and *∑A* is the sum of the integrated areas of several characteristic functional group peaks as summarized in Table 2.

### 3.4. Rheological Characterization

#### 3.4.1. Frequency Sweep Tests

Based on a standard testing procedure (AASHTO T 315-19 [26]), the complex shear modulus (G*) and phase angle (*δ*) were obtained over a wide range of temperatures and frequencies by means of DSR with oscillatory loading. In this study, DSR tests were performed using an 8-mm-diameter parallel plate with a 2-mm gap at temperatures from −10 to 30 °C and a 25-mm-diameter geometry with a 1-mm gap at temperatures from 30 to 70 °C (10 °C temperature step). The tests were carried out at a frequency sweep range from 100 to 0.1 rad/s (15.9 to 0.0159 Hz) and a strain load of 0.1%. The master curves of the complex shear modulus and phase angle at a reference temperature of 20 °C were constructed by applying the time–temperature superposition principle (TTSP). The TTSP-based master curves were used to evaluate the effect of lignin on bitumen performance.

#### 3.4.2. Linear Amplitude Sweep Tests

According to the standard testing procedure (AASHTO TP 101-14 [27]), a cyclic loading with linearly increasing strain amplitudes was used in the LAS test to assess the fatigue behavior of different binders [28]. The 8-mm-diameter parallel plates with a 2-mm gap were used in LAS tests. The LAS test consisted of two steps; in the first step, the rheological properties of the sample were tested using a frequency sweep test, which was designed to obtain information about the rheological properties. The frequency sweep test was performed at 20 °C and applied oscillatory shear loading at constant amplitude over a range of loading frequencies, employing an applied load of 0.1% strain over a range of frequencies from 0.2–30 Hz, whereby data were sampled at the following 12 typical frequencies: 0.2, 0.4, 0.6, 0.8, 1.0, 2.0, 4.0, 6.0, 8.0, 10, 20, and 30 Hz. After that, the samples were tested by applying a strain sweep, in which the frequency was 10 Hz. Bitumen is more likely to experience cracking failure under cyclic loading in DSR at low values of intermediate temperature rather than at higher testing temperatures [29]. Bitumen is soft, and its response is dictated by instability flow at high temperatures. Thus, a temperature of 20 °C was chosen to perform LAS tests to evaluate the fatigue performance of studied materials. At the selected temperature, continuous oscillatory strain-controlled cycles with linearly increasing strain amplitudes from 0% to 30% were applied to accelerate the fatigue damage of bitumen.

The fatigue resistance was then calculated based on the frequency sweep and the amplitude sweep results, as shown in Equations (3)–(6). The damage accumulation, *D(t)*, of the studied binders with testing time, *t*, can be expressed as follows:

(3) $$D\left(t\right)≅\sum_{t=1}^{N}{\left[\pi \gamma_{0}^{2}(C_{i-1}-C_{i})\right]}^{\frac{\alpha }{1+\alpha }}{\left(t_{i}-t_{i-1}\right)}^{\frac{1}{1+\alpha }}$$

where $C\left(t\right)=\frac{\left|G^{*}\right|\left(t\right)}{{\left|G^{*}\right|}_{initial}}$, $\left|G^{*}\right|\left(t\right)$ is the complex modulus at time t (MPa), ${\left|G^{*}\right|}_{initial}$ is the initial state value, *γ*_0_ is the applied strain for a given data point (%), *α* = m^−1^, in which m is the slope of an optimum-fit line in the logarithmic scale plot relating storage modulus to frequency, and *i* refers to the cycle number.

At any given time, the values of *C(t)* and *D(t)* can be obtained by fitting the relationship as follows:

(4) $$C\left(t\right)=C_{0}-C_{1}D{\left(t\right)}^{C_{2}},$$

where *C*_0_ = 1, and *C*_1_ and *C*_2_ are curve-fitting coefficients.

The damage values at failure correspond to the peak stress as follows:

(5) $$D_{f}={\left(\frac{C_{0}-C_{at\;Peak\;Stress}}{C_{1}}\right)}^{\frac{1}{C_{2}}}.$$

The fatigue parameter (*N_f_*) can be calculated as follows:

(6) $$N_{f}=A{\left(\gamma_{max}\right)}^{-B},$$

where *γ_max_* is the expected maximum strain (%), $A=\frac{f\times {\left(D_{f}\right)}^{k}}{k{\left(\pi C_{1}C_{2}\right)}^{\alpha }}$, *f* is the loading frequency (10 Hz), *k* = 1 + (1 − *C*_2_)*α*, and *B* = 2*α*.

#### 3.4.3. Relaxation Tests

The stress relaxation demonstrates the ability of a material to relieve stress under a constant strain. The studied material is intended to be applied as the surfacing layer on a pavement structure (upper layer). The relaxation tests were performed in a DSR by using a parallel-plate configuration of 8-mm diameter and 2-mm gap under strain-controlled mode at 0 °C. The tests were conducted as follows: firstly, the strain was increased from 0% to 1% shear strain in 0.1 s, and then the 1% shear strain was kept constant during a relaxation period of 200 s, while the change of shear stress was measured [30]. Longer relaxation times imply that materials are more susceptible to stress accumulation. The relaxation time should be small enough to prevent high stress accumulation in the asphalt pavement, caused by the continuous traffic load. If the stress within the pavement material does not relax sufficiently, the load of the next vehicle would accumulate more stress in the pavement.

#### 3.4.4. Glover–Rowe Parameter Tests

The location in black space diagrams (BSD) at low temperatures is an effective performance indicator to assess the cracking vulnerability of asphalt pavement materials [31]. The initial quality of bitumen as determined in the black space is an important performance indicator that can be successfully applied together with the complex modulus and phase angle to assess the aging effect on bitumen [32]. In addition, the black space diagrams could be useful for comparing the various proposed damage parameters. Based on results of the angle frequency (ω), the complex modulus (G*), phase angle (*δ*), and the dynamic viscosity (*η’*), a damage curve in black space can be built as follows:

(7) $$\frac{G^{\prime }}{\frac{\eta^{\prime }}{G^{\prime }}}=G\times \left(\frac{\cos\delta^{2}}{\sin\delta }\right)\omega .$$

Given a black space function as defined by the Glover–Rowe (G–R) parameter, an aged sample can be tested to assess the degree of damage without imposing a rigid single test temperature and frequency. Material damage due to aging is initiated when the ductility is below 5 cm, and cracking is serious when ductility reaches 3 cm [33]. The parameters were measured at a temperature of 15 °C and a frequency of 0.005 rad/s. A failure curve in the black space represents the onset of cracking as follows:

(8) $$G\times \left(\frac{\cos\delta^{2}}{\sin\delta }\right)=180\;\mathrm{kPa}.$$

Surface cracking is observed when the ductility falls to 3 cm, and the relative value of the Glover–Rowe parameter is represented by

(9) $$G\times \left(\frac{\cos\delta^{2}}{\sin\delta }\right)=450\;\mathrm{kPa}.$$

These two equations provide a damage zone in black space diagrams.

## 4. Results and Discussion

### 4.1. Microstructural Observation

**Figure 2:**
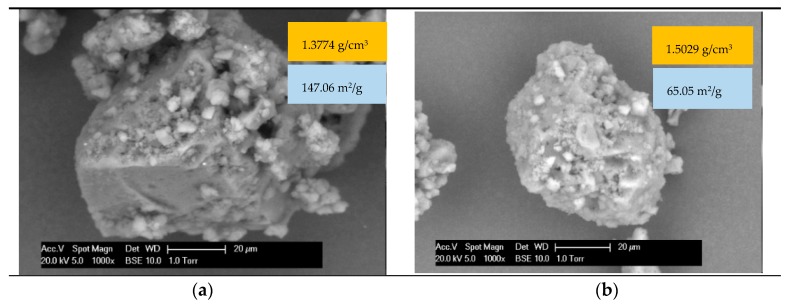
ESEM analysis results: ×1000 images of (**a**) fresh and (**b**) aged lignin.

As shown in Figure 2, lignin contains smaller fractions of particles that seem to be crushed from larger ones. The size of lignin particles ranged from 10 to 200 μm. Moreover, the fresh lignin particles had some angularity, and the surface of them was rough. The specific surface area of fresh (unaged) lignin was two times more than that of aged lignin. Generally, a finer powder results in a more irregular particle shape, a rougher surface of the particle, a more complex particle structure, and a larger area. A larger powder area results in greater friction between the particles. After aging, the density of lignin increases, and its specific surface area decreases. In addition, the density of lignin increases and its surface becomes smoother, because it contacts oxygen at high temperatures during aging.

**Figure 3:**
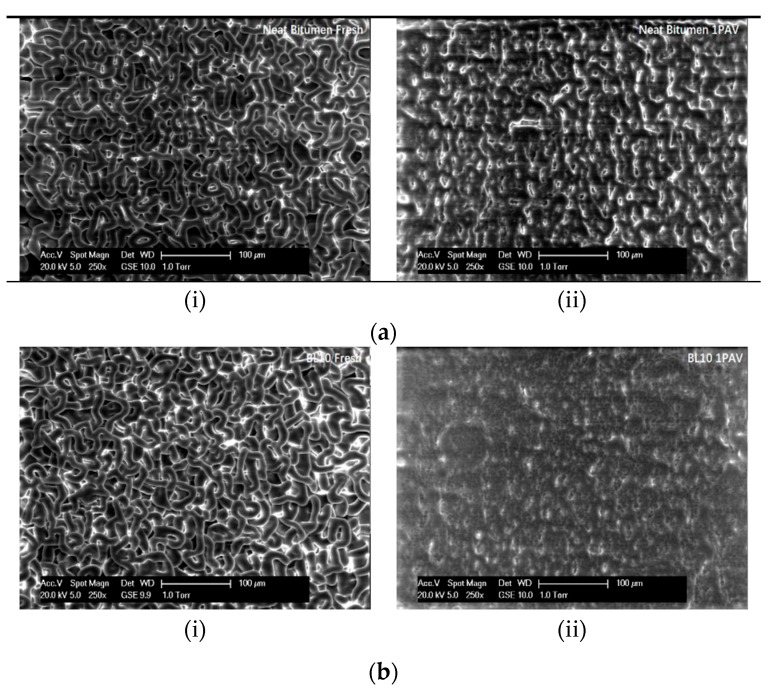
ESEM analysis results: ×1000 images of (**a**) neat bitumen and (**b**) lignin-modified binders ((i) fresh state and (ii) pressure aging vessel (PAV) aged state).

Regarding the microstructural morphology of lignin-modified bitumen, the worm structures of fresh and aged binders (i.e., Bref_F, Bref_A, BL10, and BL10 A; see Table 1) were obtained by ESEM, as shown in Figure 3a,b. In particular, all fresh binders had a relatively clear slim worm structure. The structures of the binders changed significantly after PAV aging. The density of the worm structure increased, and the thickness of the worm structure substantially increased. For the long-term aged samples, it was difficult to observe the worm structure, and a longer exposure time was needed in the gaseous secondary electron detector (GSE) mode. Moreover, no significant changes were observed with the addition of lignin. In other words, the lignin particles were not embedded in the worm structure of bitumen. It may require a longer exposure time for the lignin-modified binders to display the same worm structures.

### 4.2. Chemical Characterization

**Figure 4:**
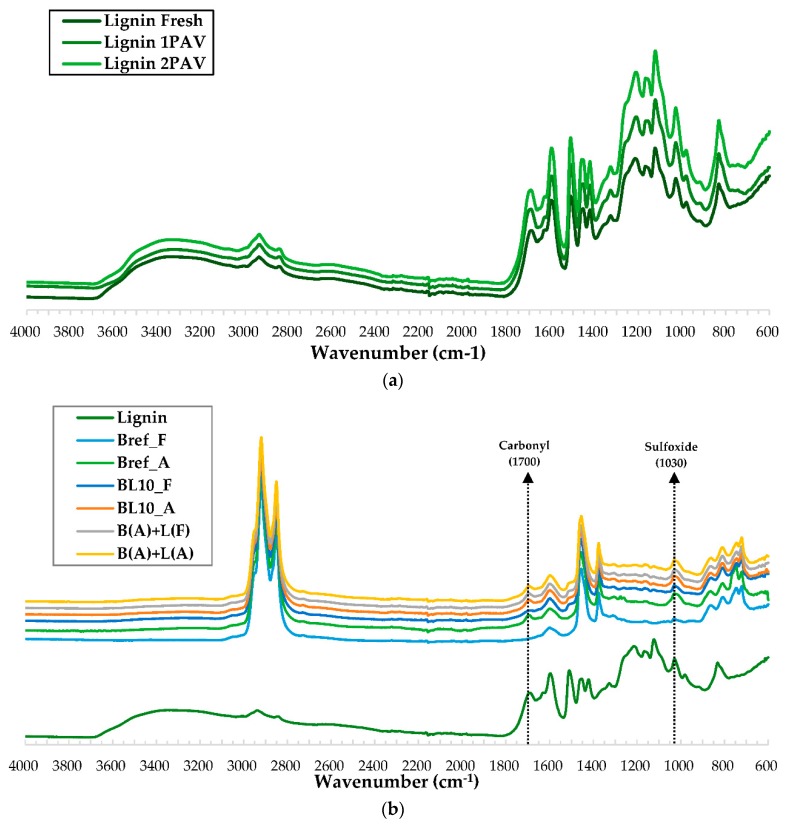
Fourier-transform infrared (FTIR) spectra of (**a**) lignin in different aging conditions, and (**b**) lignin, bitumen, and lignin-modified bitumen.

In order to understand the aging of lignin itself, lignin samples were aged in different conditions: lignin in a fresh state without aging, and aged following one and two instances of PAV, for 20 h and 40 h, respectively, after conditioning lignin powder in the oven for 2 h at 163 °C. The FTIR spectral results of lignin in different aging conditions are shown in Figure 4a, demonstrating the functional groups of lignin. Each FTIR spectrum was the average result of the nine replications. The peak values of the curves were slightly different, but the peak positions of the curves were basically the same. This shows that the aged lignin did not produce new chemical functional group peaks. Additionally, the functional group absorbance index was used to measure the number of individual chemical components in bitumen.

The typical absorbance indices are shown in Table 3. The values and the standard deviation of indices were the averages of the nine measurements. Most of the functional groups did not change or negligibly changed after the aging of lignin. This was illustrated by comparing the values of aging indices at different aging conditions. For example, the reduction of the hydroxyl group (3420 cm^−1^) was mainly due to the volatilization of water in the short-term aging process and the reaction of hydroxide in the whole aging process. The carbonyl group (1708 cm^−1^) increased due to oxidation. Most of the functional groups of lignin did not change with aging.

The change in chemical composition of different aged lignin–bitumen systems is plotted in Figure 4b. The functional group peaks of lignin-modified bitumen were determined one by one through comparing the FTIR spectra of lignin, neat bitumen (Bref_F), and lignin-modified bitumen (BL10_F) in Figure 4b. It was determined that mixing lignin and bitumen does not cause a chemical reaction, because no new functional group peaks were produced in the FTIR spectra.

**Figure 5:**
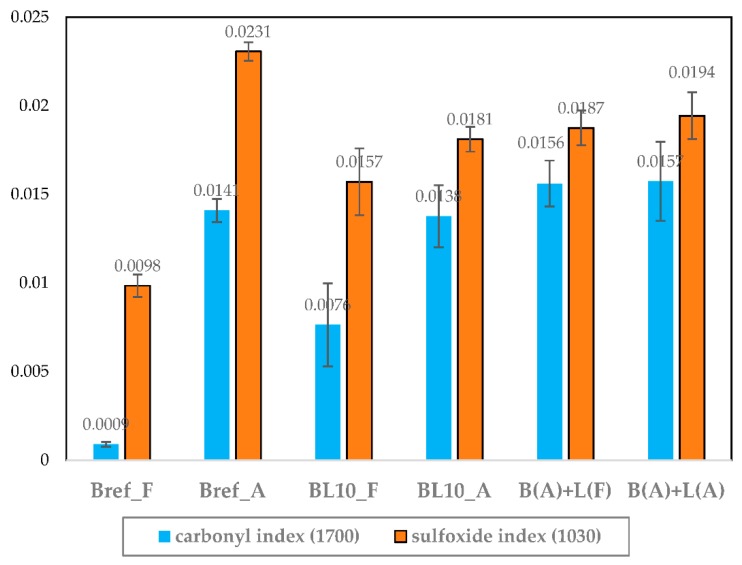
Carbonyl (1700 cm^−1^) and sulfoxide (1030 cm^−1^) indices of studied materials.

The aging indices of the studied materials were calculated, and they are provided in Figure 5. In Figure 5, the largest difference between the B(A) + L(F) and B(A) + L(A) binders was observed when aged lignin was added, indicating that the aging of lignin itself has little effect on the aging index of the whole system. The main reason was that lignin aged slightly, as shown in Table 3. In addition, the lignin content in lignin-modified bitumen was only 10% by mass of bitumen. The aging effect of bitumen was more obvious. Therefore, the difference between the fresh and aged lignin can be ignored. Carbonyl and sulfoxide aging indices can still be used to quantify the aging state of lignin-modified bitumen.

Both carbonyl and sulfoxide indices increased with aging. The fresh and aged virgin bitumen (Bref_F and Bref_A) were compared to indicate the aging extent of binders without lignin. Upon comparing Bref_A and BL10_A, it can be seen that the two materials (lignin and bitumen) were mixed firstly and then aged, which did reduce the rate of aging. Carbonyl and sulfoxide indices of neat bitumen increased from 0.0009 and 0.0098 to 0.0141 and 0.0231, respectively. However, for the BL10_F and BL10_A, the aging indices increased from 0.0076 and 0.0157 to 0.0138 and 0.0181 because lignin was added before aging. Comparing to BL10_A and B(A) + L(A), in which lignin and bitumen were aged separately and then mixed together, produced more aging functional groups.

Lignin was added and mixed uniformly in the bitumen. Lignin particles precipitate in bitumen. During the aging process of neat bitumen, the surface bitumen is exposed to the external environment, including temperature and air, and it would age first. As oxygen diffuses into the bitumen, the internal bitumen starts aging. For bitumen mixed with lignin particles, when oxygen enters the interior, it is necessary to bypass the obstacle formed by the lignin particles and the bituminous film. It would take a long time for oxygen in the air to enter the bitumen due to the entry path increasing. Therefore, the contact time between the bitumen and oxygen is reduced, and the oxidation effect of the bitumen is inhibited. Since the lignin particles precipitate in bitumen, the loss of light components in the bitumen is correspondingly reduced. This also delays the accumulation of asphaltenes. Overall, bitumen and lignin should be mixed together firstly and then aged to maximize the anti-oxidation effect of lignin in bitumen.

### 4.3. Rheological Characterization

#### 4.3.1. Frequency Sweep Tests

**Figure 6:**
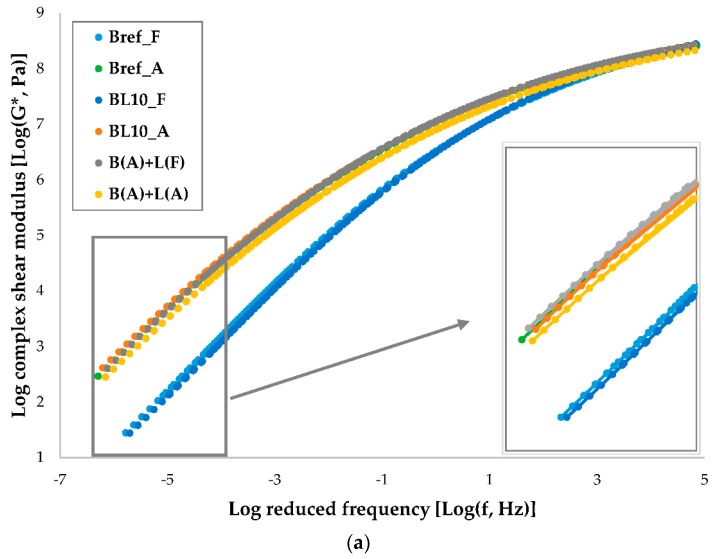

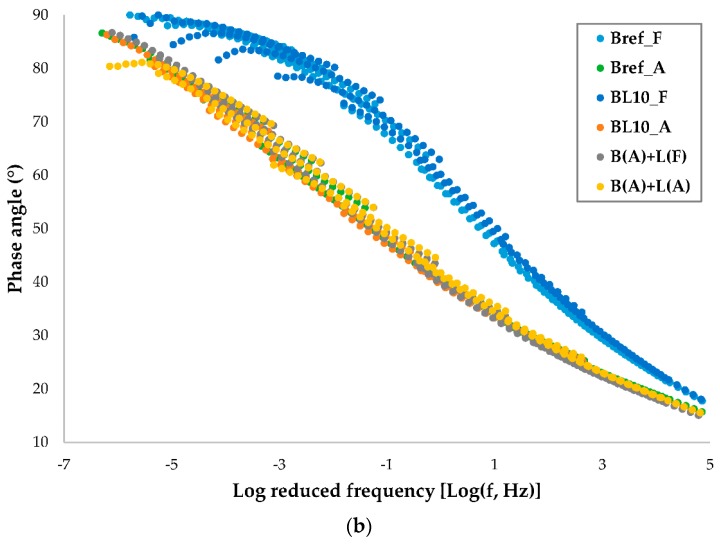
Master curves of (**a**) complex shear modulus and (**b**) phase angle.

The master curves of complex modulus and phase angle of different materials are shown in Figure 6. A higher modulus of the material indicates a stronger resistance to deformation. A lower phase angle means that the material is more elastic and that the delay in response between stress and strain is shorter. Each tested material had three replicates. Obviously, the complex modulus increased, and the phase angle decreased with aging. In the low-temperature region, the properties of the samples were very similar and stable in terms of modulus and phase angle. However, the effect of lignin on bitumen was mainly reflected at high temperatures.

The complex modulus and phase angle results of the three materials (i.e., BL10_A, B(A) + L(F), and B(A) + L(A)) were almost identical and overlapped each other, as determined by comparing them with fresh samples. The aging of lignin itself had little effect on the rheological properties of lignin-modified bitumen, as seen by comparing B(A) + L(A) and B(A) + L(F). In addition, there was little effect on the rheological properties of adding lignin before or after aging, as seen by comparing BL10_A and B(A) + L(A).

**Figure 7:**
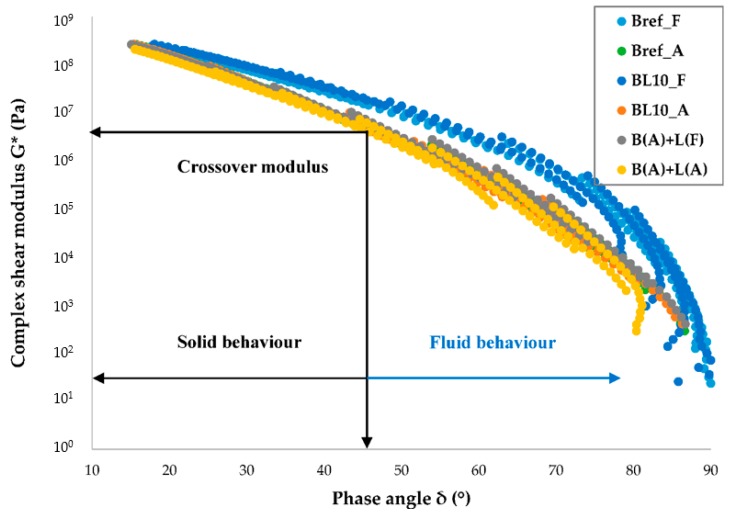
The rheological properties in a black diagram.

Additionally, to eliminate the influence of shift factor, the changes in bitumen rheology are depicted in black space diagrams [31] in Figure 7. After aging, the shape of the curve moved to a straighter curve. The decrease in phase angle and the increase in modulus denoted a tendency toward a more brittle material. It is clear from Figure 7 that, in addition to the fresh samples, the results of modified binders were quite close.

**Figure 8:**
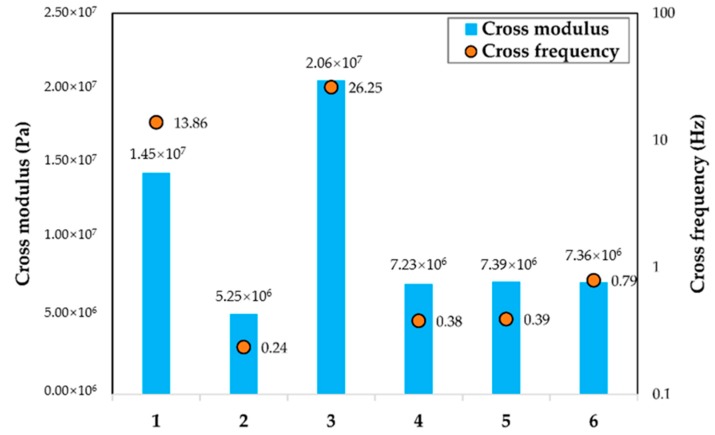
The rheological properties, crossover modulus, and crossover frequencies.

The most common method to characterize the viscoelastic fluid-to-solid transitional behavior is the crossover frequency (where the storage modulus and loss modulus are equal, i.e., the phase angle is 45°) of the storage modulus and the loss modulus [34]. The complex modulus corresponding to the crossover frequency is called the crossover modulus, which is shown in Figure 7. A lower crossover frequency reveals that bitumen has a higher molecular mass, longer relaxation time, and higher softening point, while a lower crossover modulus indicates wider molecular mass distribution and higher polydispersity [35,36]. The crossover modulus and frequencies of different samples are shown in Figure 8. It shows that aged bitumen had a lower crossover frequency and modulus. The results of aged lignin-modified bitumen were similar except for the unaged samples.

#### 4.3.2. Linear Amplitude Sweep Tests

**Figure 9:**
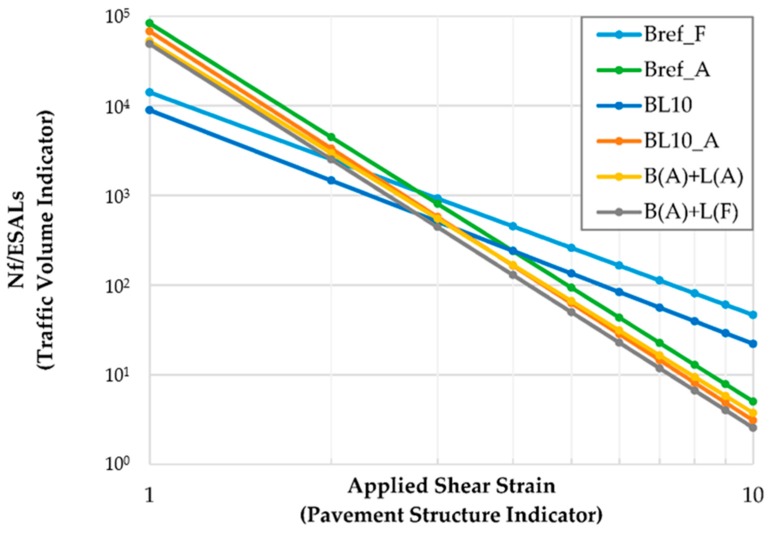
The plot of fatigue parameter *N_f_* versus applied shear strain (20 °C).

The fatigue resistance of studied materials was determined by LAS tests. Figure 9 shows the fatigue life (*N_f_*) at different strain levels after performing the calculation, as mentioned in the previous section. The equation for the fatigue lines is listed in Table 4. With the increase in strain level, a significant decrease in *N_f_* was observed. The strain level had a significant influence on the order of the fatigue life of the materials. In addition, the fatigue life performance was ranked as Bref_A to BL10_A, B(A) + L(A), B(A) + L(F), Bref_F, and BL10 at low strain levels (e.g., 2%). Bitumen becomes stiffer due to aging, and a stiffer material shows higher resistance to micro-deformation at low strain levels. In general, the addition of lignin lightly reduced the fatigue life of lignin-modified bitumen. On the other hand, the fatigue life performance at higher strain levels (e.g., 5%) was ranked as Bref_F to BL10, Bref_A, B(A) + L(A), BL10_A, and B(A) + L(F). Fresh bitumen showed a better fatigue life. Because the fresh bitumen has better viscous behavior than the aged one at high strain levels, damage is less likely to occur on the fresh bitumen. Moreover, the power of function for the fresh material was significantly larger than that of the other aged materials. Therefore, the aging process decreased the power of the fatigue function. Furthermore, the addition of lignin slightly reduced the fatigue life of bitumen, as seen by comparing the lines of Bref_F, BL10, Bref_A, and BL10_A samples. Another interesting point is that several straight lines had a similar fatigue life at 3% strain level. This point actually represents material strain sensitivity. A smaller point denotes more sensitivity. The performance at low strain levels should be emphasized. Compared to Bref_A, the other three aged samples (BL10_A, B(A) + L(A), and B(A) + L(F)) had very similar fatigue life. The aging of lignin itself and the moment that lignin was added to the bitumen had an inconspicuous effect on fatigue life. Physical interactions played a predominant role in fatigue life.

#### 4.3.3. Relaxation Tests

**Figure 10:**
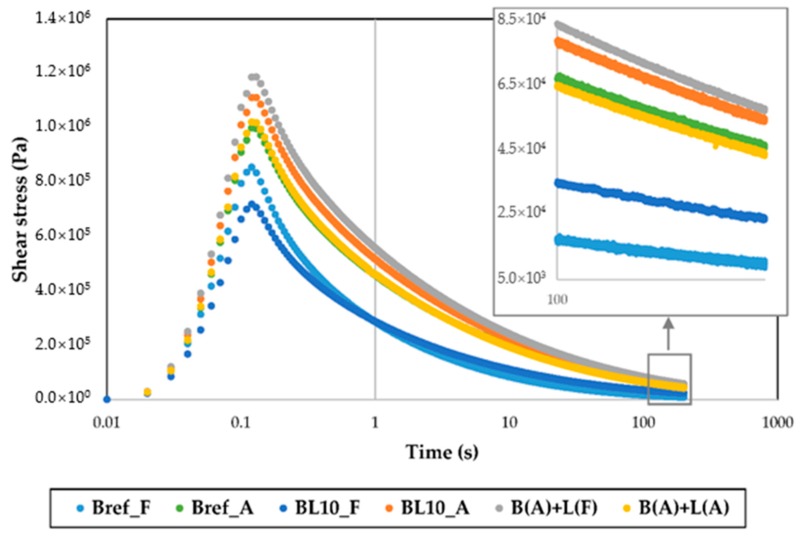
Relaxation results of the relationship between shear stress and relaxation time (at 1% shear strain and 0 °C).

**Figure 11:**
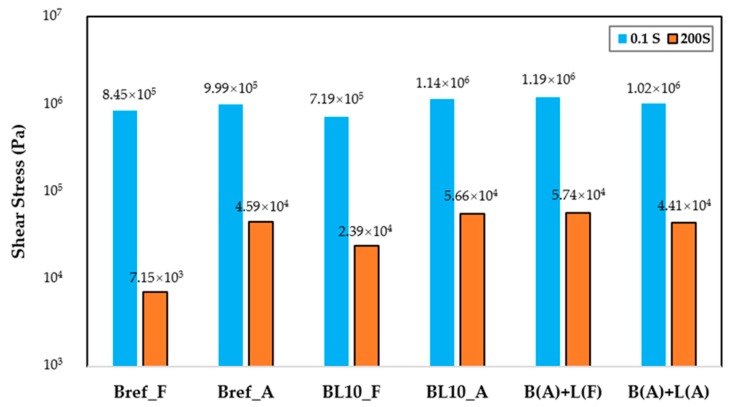
Shear stress at the initial (after 0.1 s) and end (200 s) times.

Figure 10 illustrates the change in shear stress of different studied materials with relaxation time. As materials age, the residual shear stress of aged materials increases after the same relaxation time period due to the increase in relaxation modulus [37]. Samples (BL10_A, B(A) + L(A), and B(A) + L(F)) produced using different preparation methods showed similar relaxation properties. To further evaluate the properties of these materials, the absolute value of shear stress at 0.1 s and 200 s and the ratio of residual shear stress (200 s) divided by the initial status (0.1 s) were plotted, as shown in Figure 11, depicting the stress at the initial and end times. Every sample had three replicates.

**Figure 12:**
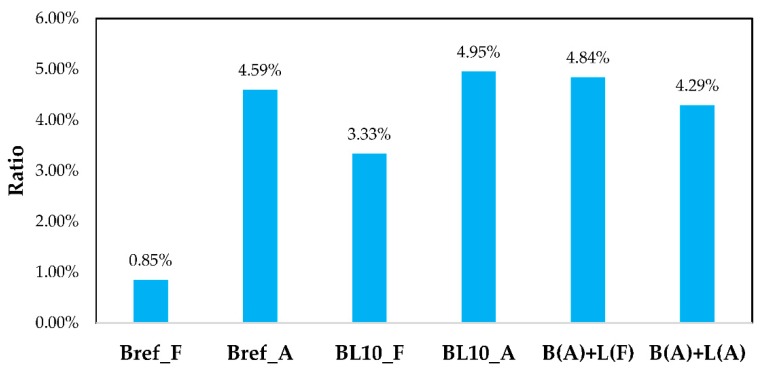
The ratio of residual shear stress (200 s) versus the initial shear stress (0.1 s).

The initial and residual shear stresses of samples following different aging processes are depicted in Figure 11. Obviously, the aged samples had higher initial and residual shear stresses compared with fresh ones. The initial and residual shear stresses increased with the aging process. Upon reaching the same strain level in a shorter time (0.1 s), a higher initial shear stress means that the material had a higher modulus. Analyzing the data of Bref_A and BL10_A together, mixing with lignin had little effect on the initial and residual shear stress. The initial shear stress of B(A) + L(A), B(A) + L(F), and BL_A was similar, and it was about 1.4 times larger than that of BL_F. After a relaxation period of 200 s, the residual shear stress of these three aged materials was twice the value of the fresh samples. Comparing different aged materials, the order of initial shear stress was from B(A) + L(F) to BL_A and B(A) + L(A). Figure 12 shows the ratio of residual shear stress versus the initial shear stress of different samples.

**Figure 13:**
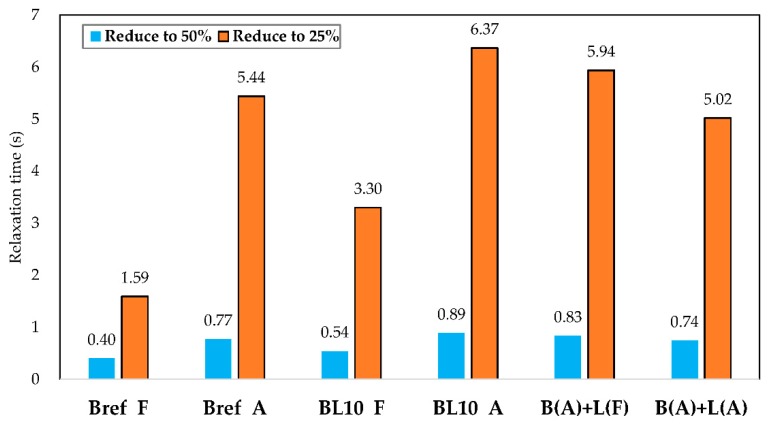
The relaxation time when the shear stress was reduced to 50% and 25% of the initial stress.

The results show that the shear stress ratio increased with aging. For the neat bitumen, 0.85% of the initial shear stress remained after 200 s of relaxation; however, 4.59% shear stress remained in the aged sample Bref_A after relaxation. The same conclusion could be obtained from BL10_F and BL10_A. A lower ratio denotes a better relaxation property. Fresh specimens showed better elasticity than aging specimens. Therefore, they showed a better recovery ability and the ratio was smaller. The addition of lignin to fresh bitumen increased the stress ratio and reduced the relaxation property. In three different combinations, lignin and bitumen mixed after aging showed the minimum stress ratio in the aged samples. Due to the fact that traffic loading is usually continuous, the relaxation time of bitumen needs to be short enough to prevent stress accumulation in the pavement. The testing load should correspond to the traffic load frequencies or load time periods. Considering the fact that the bituminous materials are viscoelastic, then this time period should be linked to the recovery phase after loading, which subsequently affects the stress and, thus, the damage accumulation in the material. The relaxation time, as the shear stress was reduced to 50% and 25% of the initial stress, is depicted in Figure 13.

Figure 13 indicates that the relaxation time increased after aging when the shear stress was reduced to 50% and 25% of the initial stress. The viscosity of samples increased with aging, contributing to the relaxation time increase. For fresh materials, the shear stress of Bref_F reduction to 25% needed 1.59 s and that of BL10_F needed 3.30 s. However, for the other aged samples (Bref_A, BL_A, B(A) + L(F), and B(A) + L(A)) 5.44, 6.37, 5.94, and 5.02 s were required, respectively. When the lignin was added in advance, the relaxation time reduced to 50% and 25% when aging was increased (see BL_A with B(A) + L(A)). In summary, the aged sample had higher shear stress at initial and end times, a higher ratio of residual stress, and a longer relaxation time than the fresh sample. In addition, the relaxation properties of BL_A, B(A) + L(A), and B(A) + L(F) were similar compared to the fresh samples. The addition of lignin did not improve the relaxation properties dramatically. However, the aging of lignin itself and the moment that lignin was added to the bitumen had an effect on the relaxation properties, especially the relaxation ratio of residual and initial shear stress and the relaxation time when reducing to certain stress levels.

#### 4.3.4. Glover–Rowe Parameter Tests

**Figure 14:**
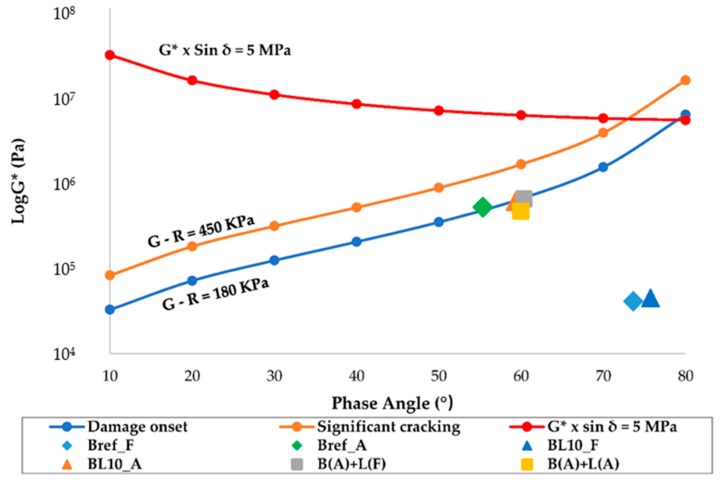
Glover–Rowe damage parameter in black space diagrams.

The shape of the Glover–Rowe (G–R) curve and the current Superpave fatigue parameter (G* × sin*δ* = 5 MPa) were different. Based on the data of the samples, the curve shape of G* × sin*δ* is not a logical damage indicator. The test conditions (15 °C, 0.005 rad/s) should be used for frequency sweeps to assess failure performance. Thus, the results of the Glover–Rowe damage parameter are shown in Figure 14. The two lines (G–R = 180 kPa; G–R = 450 kPa) provide the damage zone in the black space diagram. The red line shows the limit of the current Superpave fatigue parameter (G* × sin*δ* = 5 MPa). The rhombuses, triangles, and squares with different colors denote different samples. Fresh specimens were in a very safe state. The properties approached the damage zone as aging proceeded. Based on the calculated data, the values of several aged samples were close to the damage onset line, but they did not enter the damage zone except for Bref_A. During the aging process, cracking began in the neat bitumen. However, with the addition of lignin, the time for the crack to appear was delayed. Lignin is, thus, beneficial for cracking resistance properties. Three samples (BL10_A, B(A) + L(A), and B(A) + L(F)) showed extremely close values and properties. This indicates that, independently of whether lignin ages or not, the adding process has little effect on the cracking resistance of the material.

## 5. Conclusions

Lignin was added to bitumen to evaluate the interaction between the two materials after aging. Based on the current preliminary results, the main conclusions are as follows:

1. After aging, the specific surface area of lignin particles decreases. The microstructure of bitumen with and without lignin is almost the same. However, it becomes difficult to observe the worm structure of bitumen after aging and the addition of lignin.
2. The results from FTIR tests show that the various functional groups of lignin do not change remarkably during aging, and carbonyl and sulfoxide indices can still be used to assess the aging state of lignin-modified bitumen.
3. The effect of lignin added in advance or after aging has little effect on the viscoelastic characteristics of bitumen. The physical interaction between lignin and bitumen plays an important role, as shown by the changes in complex modulus, phase angle, crossover modulus, and crossover frequency.
4. The addition of lignin slightly reduces the fatigue life based on the results of LAS tests. As bitumen ages, its fatigue life increases at low strain levels and decreases at high strain levels due to the stiffening effect.
5. The addition of lignin does not improve the relaxation properties dramatically. However, the aging of lignin itself and the moment that lignin is added to the bitumen has an effect on the relaxation properties, especially the relaxation ratio of residual and initial shear stress and the relaxation time to certain stress levels.
6. Based on the results calculated by the Glover–Rowe parameter method, lignin provides an improvement in cracking resistance. Nevertheless, the aging of lignin itself and the moment that lignin is added to the bitumen have little impact on the cracking sensitivity of bitumen.

Overall, the addition of lignin has some positive effects as an anti-oxidant in bitumen. In this study, only a dosage of 10% by mass of bitumen, which was determined elsewhere, was used. Other lignin contents and bitumen types will be compared to verify the above conclusions. The compatibility between lignin and bitumen due to the differences in structure and density will be researched further, including the separation after blending and storage modulus.

## Figures and Tables

**Table 1:** Studied materials.

Studied Materials	Modification by Bitumen Mass	Explanation
Bref_F	0%	Fresh neat bitumen, as reference
Bref_A		Aged neat bitumen
BL10_F	10%	Fresh bitumen mixed with fresh lignin
BL10_A		Fresh bitumen mixed with fresh lignin, then aging
B(A) + L(F)		Aged bitumen mixed with fresh lignin
B(A) + L(A)		Bitumen and lignin aged separately, then mixing

**Table 2:** Main functional groups of lignin (*^1^) and bitumen (*^2^) in Fourier-transform infrared (FTIR) spectra.

Wavenumber (cm^−1^)	Function Groups
3500–3100	Stretching vibrations of –OH groups *^1^
1753–1660	Stretching vibrations of C=O bonds *^1^
1620–1555	Vibrations of aromatic ring *^1^
1525–1480	Vibrations of aromatic ring *^1^
1280–1245	Stretching vibrations of C–O bonds *^1^
1170–1140	Deformation vibrations of C–H bonds in guaicyl rings *^1^
1140–1100	Deformation vibrations of C–H bonds in syryngyl rings *^1^
1095–1070	Deformation vibrations of C–O bonds in secondary alcohols and aliphatic ethers *^1^
1070–995	Deformation vibrations of C–H bonds in the aromatic rings and C–O bonds in primary alcohols *^1^
2990–2880	Stretching aromatic *^2^
2880–2820	Stretching symmetric *^2^
1753–1660	Oxygenated functional group (carbonyl) *^2^
1670–1535	Aromatic structures *^2^
1525–1395	Aliphatic structures *^2^
1390–1350	Branched aliphatic structures *^2^
1047–995	Oxygenated functional group (sulfoxide) *^2^
912–838	Out of singlet *^2^
838–783	Out of adjacent *^2^
783–734	Out of adjacent *^2^
734–710	Long chains *^2^

**Table 3:** The typical functional group absorbance index for lignin. PAV—pressure aging vessel.

Age State	Items	Absorbance Band Values (cm^−1^)								
		3420	1708	1597	1512	1269	1150	1125	1085	1032
Fresh	Value	0.2900	0.1640	0.1800	0.1772	0.0240	0.0095	0.0753	0.0054	0.0747
	SD	0.0189	0.0098	0.0033	0.0033	0.0022	0.0010	0.0114	0.0004	0.0068
1 PAV	Value	0.2736	0.1796	0.1833	0.1824	0.0252	0.0120	0.0652	0.0053	0.0734
	SD	0.0177	0.0050	0.0030	0.0040	0.0015	0.0011	0.0053	0.0007	0.0050
2 PAV	Value	0.2746	0.1770	0.1877	0.1813	0.0259	0.0123	0.0650	0.0056	0.0707
	SD	0.0191	0.0049	0.0035	0.0035	0.0024	0.0012	0.0065	0.0005	0.0056

**Table 4:** Fatigue lines of studied materials.

Studied Materials	$\left(N_{f}=A{\left(\gamma \right)}^{-B}\right)$
Bref_F	$N_{f}=14134{\left(\gamma \right)}^{-2.4832}$
Bref_A	$N_{f}=83264{\left(\gamma \right)}^{-4.2192}$
BL10_F	$N_{f}=8946{\left(\gamma \right)}^{-2.6073}$
BL10_A	$N_{f}=67739{\left(\gamma \right)}^{-4.3408}$
B(A) + L(A)	$N_{f}=52522{\left(\gamma \right)}^{-4.1466}$
B(A) + L(F)	$N_{f}=48997{\left(\gamma \right)}^{-4.2816}$
